# Molecular phylogeny and taxonomy of the genus *Nectogale* (Mammalia: Eulipotyphla: Soricidae)

**DOI:** 10.1002/ece3.9404

**Published:** 2022-10-13

**Authors:** Ronghui Fan, Keyi Tang, Liang Dou, Changkun Fu, Abu ul Hassan Faiz, Xuming Wang, Yufan Wang, Shunde Chen, Shaoying Liu

**Affiliations:** ^1^ College of Life Sciences Sichuan Normal University Chengdu China; ^2^ Museum of Natural History/School of Life Sciences, Key Laboratory of Bio‐Resources and Eco‐Environment of Ministry of Education, Key Laboratory of Conservation Biology on Endangered Wildlife of Sichuan Province Sichuan University Chengdu China; ^3^ Department of Zoology Women University of Azad Jammu and Kashmir Bagh Pakistan; ^4^ Sichuan Academy of Forestry Chengdu China; ^5^ Nature of East China Zhejiang China

**Keywords:** mitochondrial DNA, molecular phylogeny, *Nectogale*, nuclear DNA, taxonomy

## Abstract

The elegant water shrew, *Nectogale elegans*, is one of the small mammal species most adapted to a semi‐aquatic lifestyle. The taxonomy of the genus *Nectogale* has received little attention due to difficulties in specimen collection. In this study, we sequenced one mitochondrial and eight nuclear genes to infer the phylogenetic relationship of *Nectogale*. Phylogenetic analyses revealed two large clades within *Nectogale*. One clade represented *N. elegans*, and the other was regarded as *N. sikhimensis*. The split between *N. elegans* and *N. sikhimensis* dated back to the early Pleistocene (2.15 million years ago [Ma]), which might be relevant to the Qinghai‐Tibet Plateau (QTP) uplift. The morphological comparison showed several distinguishing characters within *Nectogale*: the shape of the mastoids, the first lower unicuspid (a1), and the second upper molar (M^2^). Overall, the molecular and the morphological evidences supported that the genus *Nectogale* consists of two valid species: *N. elegans* and *N. sikhimensis.*

## Graphical abstract

We recover species status of *Nectogale sikhimensis* using molecular and morphometric analyses, and the genus Nectogale consists of two valid species, *N. elegans* and *N. sikhimensis*.

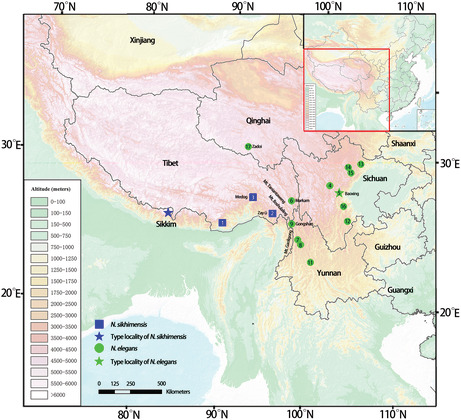

## 1 INTRODUCTION

It has been widely accepted that the Soricinae is divided into six tribes (Burgin & He, 2018; Dubey et al., 2007; Hutterer, 2005), and Nectogalini is one of them. Three of the six genera of tribe Nectogalini are adapted to a semi‐aquatic lifestyle (*Nectogale* Milne ‐Edwards, 1870; *Neomys* Kaup, 1829; and *Chimarrogale* Anderson, 1877) (Burgin & He, 2018; Hutterer, 1985). The elegant water shrew, *Nectogale elegans*, is the only known species in the genus *Nectogale*, which was generally considered to be the most adapted species in the family to live in water (Smith & Xie, 2009). These large‐sized insectivores are externally characterized by small eyes, obviously reduced external ears, and large and webbed feet and are distributed in southwestern China, Nepal, Sikkim (India), Bhutan, and northern Myanmar (Jiang, 2015; Smith & Xie, 2009; Wang & Hu, 1999).

In 1870, Milne–Edwards described the first species, *N. elegans*, based on specimens collected from Mouping (=Baoxing), in northwestern Sichuan, China. Subsequently, Winton (1899) examined two specimens of *Nectogale* from Sikkim and nominated them *N. sikhimensis* (Winton, 1899) based on morphological differences with *N. elegans*. He considered the two specimens browner in color without a clear boundary between dorsal and ventral pelage, and the shorter cusps of the first upper incisor. However, Allen believed that this should be considered a subspecies (Allen, 1938), and this was followed by some scholars (Ellerman & Morrison‐Scott, 1951; Smith & Xie, 2009; Wang, 2003). On the other hand, *N. sikhimensis* has been treated by other scholars as conspecific or synonym of *N. elegans* (Hoffmann, 1987; Hutterer, 1993, 2005; Paradiso, 1975; Repenning, 1967).

The phylogenetic relationship of this genus remains to be studied due to the lack of specimens of *N. sikhimensis* in previous studies based on morphological (Hoffmann, 1987) and phylogenetic analyses (He et al., 2010; Ohdachi et al., 2006). Is the *N. sikhimensis* a taxonomically valid species? In this study, we collected specimens of *Nectogale* species from Sichuan, Yunnan, Qinghai, and Tibet in China, and used one mitochondrial and eight nuclear genes to infer the phylogenetic relationship, estimated the divergence time, and explored the evolutionary history of *Nectogale*.

## 2 MATERIALS AND METHODS

### 2.1 Sampling and DNA sequencing

**FIGURE 1:**
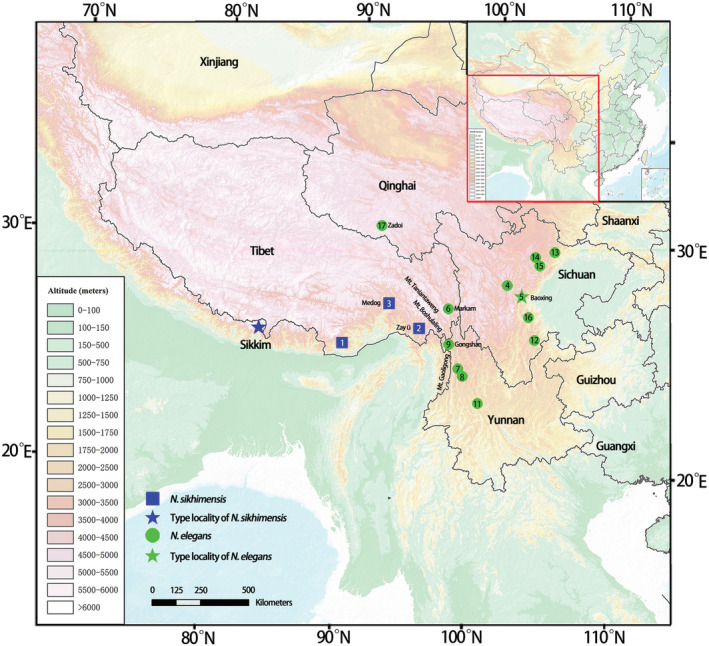
Sample localities of specimens used in the present study.

A total of 44 specimens were collected from Sichuan, Yunnan, and Tibet, including 42 individuals of *Nectogale* and two of *Chimarrogale* (Figure 1 and Table 1). Two individuals from *C. styani* (Winton, 1899) were used as outgroups for phylogenetic analyses because of the close phylogenetic relationship between the genera *Chimarrogale* and *Nectogale* (He et al., 2010; Ohdachi et al., 2006). All specimens were identified based on their morphology and distributions following de Winton and Styan (1899), Smith and Xie (2009), and Wang (2003). In addition, some of the other sequences used in molecular analyses were downloaded from GenBank (Tables S1 and S2). The voucher and museum number, location data, and elevation are provided in Table S1.

**TABLE 1:** Information of samples used in this study

Species	Site	Voucher/accession number	Localities	Longitude	Latitude	Elevation (m)	Source
*N. sikhimensis*	1	AB175095	Southern Tibet	–	–	–	GenBank
	2	CSD402	Zayü, Tibet	97.017	28.500	1550	This study
	2	CSD717	Zayü, Tibet	97.017	28.500	1550	This study
	2	CSD4601	Zayü, Tibet	–	–	–	This study
	3	CSD877	Medog, Tibet	95.333	29.325	–	This study
	3	CSD880	Medog, Tibet	95.333	29.325	–	This study
	3	CSD1507	Medog, Tibet	95.333	29.325	2160	This study
	3	CSD4295	Medog, Tibet	95.333	29.325	–	This study
*N. elegans*	4	CSD5233	Danba, Sichuan	101.890	30.878	1900	This study
	5	FT142	Baoxing, Sichuan	102.915	30.533	–	This study
	5	FT143	Baoxing, Sichuan	102.915	30.533	–	This study
	5	FT144	Baoxing, Sichuan	102.915	30.533	–	This study
	5	KC503902	Baoxing, Sichuan	102.729	30.847	–	GenBank
	6	CSD3235	Markam, Tibet	98.351	29.617	2600	This study
	6	CSD3236	Markam, Tibet	98.351	29.617	2600	This study
	7	CSD4467	Lanping, Yunnan	99.381	26.358	1750	This study
	7	CSD4468	Lanping, Yunnan	99.381	26.358	1750	This study
	7	CSD4469	Lanping, Yunnan	99.381	26.358	1750	This study
	7	CSD4470	Lanping, Yunnan	99.381	26.358	1750	This study
	7	CSD4471	Lanping, Yunnan	99.381	26.358	1750	This study
	7	CSD4472	Lanping, Yunnan	99.381	26.358	1750	This study
	7	CSD4473	Lanping, Yunnan	99.381	26.358	1750	This study
	8	CSD5036	Lanping, Yunnan	99.226	26.501	2270	This study
	8	CSD5037	Lanping, Yunnan	99.226	26.501	2270	This study
	8	CSD5038	Lanping, Yunnan	99.226	26.501	2270	This study
	8	CSD5039	Lanping, Yunnan	99.226	26.501	2270	This study
	8	CSD5040	Lanping, Yunnan	99.226	26.501	2270	This study
	8	CSD5041	Lanping, Yunnan	99.226	26.501	2270	This study
	8	CSD5042	Lanping, Yunnan	99.226	26.501	2270	This study
	8	CSD5043	Lanping, Yunnan	99.226	26.501	2270	This study
	8	CSD5044	Lanping, Yunnan	99.226	26.501	2270	This study
	9	GU981291	Gongshan, Yunnan	98.332	27.937	–	GenBank
	9	GU981292	Gongshan, Yunnan	98.349	27.736	–	GenBank
	10	GU981293	Jingdong, Yunnan	100.642	24.422	–	GenBank
	11	GU981294	Nanjian, Yunnan	100.491	24.912	–	GenBank
	12	HX‐01‐01	Meigu, Sichuan	103.132	28.328	–	This study
	12	HX‐01‐02	Meigu, Sichuan	103.132	28.328	–	This study
	12	HX‐01‐03	Meigu, Sichuan	103.132	28.328	–	This study
	12	HX‐01‐04	Meigu, Sichuan	103.132	28.328	–	This study
	12	HX‐01‐05	Meigu, Sichuan	103.132	28.328	–	This study
	12	HX‐01‐06	Meigu, Sichuan	103.132	28.328	–	This study
	12	HX‐01‐08	Meigu, Sichuan	103.132	28.328	–	This study
	12	MGLL‐12‐01	Meigu, Sichuan	103.132	28.328	–	This study
	13	930,182	Maoxian, Sichuan	103.852	31.681	–	This study
	14	GX02070501	Lixian, Sichuan	103.167	31.436	1570	This study
	14	GX02070502	Lixian, Sichuan	103.167	31.436	1570	This study
	15	Jinbo604	Wenchuan, Sichuan	103.389	31.209	1800	This study
	16	PXG070709	Tianquan, Sichuan	102.758	30.066	2600	This study
	17	MN535080	Zadoi, Qinghai	95.301	32.893	–	GenBank
*C. styani*	7	CSD4476	Lanping, Yunnan	99.381	26.358	1750	This study
*C. styani*	7	CSD4477	Lanping, Yunnan	99.381	26.358	1750	This study

Total genomic DNA was extracted from the muscle or the liver tissue preserved in 95% ethanol using the phenol/proteinase K/sodium dodecyl sulfate method (Sambrook et al., 1989). All muscle or liver tissues were stored in 100% ethanol at −70°C after DNA extraction for further analysis. We amplified one mitochondrial gene (complete cytochrome b [CYT B, 1128 bp]) from all samples (except two *Chimarrogale styani*) and eight nuclear genes (recombinant adenosine a3 receptor [ADORA3, 351 bp], brain‐derived neurotrophic factor [BDNF, 534 bp], von Willebrand factor [VWF, 864 bp], adenosine triphosphate 7a [ATP7A, 663 bp], growth hormone receptor [GHR, 768 bp], administered beta 2 [ADRB2, 804 bp] and recombination activating 2 [RAG2, 687 bp], and breast cancer susceptibility gene 1 [BRCA1, 384 bp]). These genes represented good genetic markers to solve the molecular phylogenetics of some mammals (He et al., 2017; Meredith et al., 2011). PCR amplifications were performed in a reaction volume mixture of 25 μl, containing 3 mM MgCl_2_, 0.2 U rTaq Polymerase (Takara, Dalian, China), 1× reaction buffer, 0.2 mM of each dNTP, 0.4 mM of each primer, and approximately 100–500 ng of genomic DNA. The primers used are listed in Table S3. PCR conditions consisted of an initial denaturing step at 94°C for 5 min, followed by 40 cycles of 45 s denaturation at 94°C, 45 s annealing at 49°C (58°C, all nuclear genes), 90 s extension at 72°C, and a final extension step at 72°C for 10 min. The PCR products were then electrophoresed on a 1% agarose gel, visualized with ethidium bromide staining to verify PCR quality, and purified using ethanol precipitation. The purified PCR products were directly sequenced with both sense and anti‐sense primers using the Big Dye terminator kit and determined using an ABI 310 analyzer (Applied Biosystems).

### 2.2 Molecular data processes and analyses

All sequences were edited using EditSeq (DNASTAR, Lasergene v7.1) and aligned using MEGA5 (Tamura et al., 2011). Two methods were used to infer phylogenetic relationships: Bayesian Inference (BI) and maximum likelihood (ML). All sequences were divided into two datasets: (1) an eight nuclear gene combined dataset (nDNA); (2) a CYT B gene dataset (mtDNA), including seven sequences of *Nectogale* and two of *C. styani* downloaded from GenBank (Table S1). The sequences of *C. styani* were used as outgroups. Bayesian gene trees were reconstructed independently for the CYT B gene and concatenated nDNA fragments. The Bayesian analysis was performed with BEAST v1.6.1 (Drummond et al., 2012). Each gene was treated as a partition. The jModelTest 2.1.0 (Darriba et al., 2012) was used to determine the best fitting model for each gene under the Akaike information criterion (AIC). The best fitting models were as follows: (1) HKY for ADORA3, VWF, GHR, and BRCA1; (2) HKY + I for ADRB2; (3) HKY + G for CYT B; (4) GTR for BDNF and RAG2; and (5) GTR + I + G for ATP7A. The analysis used unlinked substitute models, linked clock models, linked trees, an uncorrected lognormal, a relaxed molecular clock model, a birth‐death tree prior, and default prior (including a random starting tree). Each analysis ran for 100 million generations and was sampled every 5000 generations. We repeated the analysis 10 times and evaluated the convergence using Tracer 1.5 (Drummond et al., 2012). Posterior probabilities (PP) > 0.95 were considered to be strongly supported (Huelsenbeck & Bruce, 2004). For ML analyses, we used RAxML v8.2.12 (Stamatakis, 2014) on the CIPRES Science Gateway v3.3 (http://www.phylo.org) (Miller et al., 2010). The ML analyses used partitioned datasets (each gene as a partition) and chose the GTRGAMMA model for bootstrapping phase. The analyses used the fast bootstrapping algorithm with 500 replicates.

In addition, we calculated the average genetic distance between the clades. All calculation results were based on within and between group pairwise analysis using the Kimura 2‐Parameter (K2P) model in MEGA5, with 1000 bootstrap replications (Tamura et al., 2011).

We estimated the divergence time using BEAST v1.7.5. Because mitochondrial genes may overestimate the true divergence time (Phillips, 2009; Zheng et al., 2011), the combined nDNA genes dataset was used for the estimation of divergence times. All calibration age constraints were treated as log‐normal distributions. We used three calibration points: (1) The division of Soricinae and Crocidurinae occurred at 36 million years ago (Springer et al., 2018). So, we set the mean = 36 and stdve = 0.135 (He et al., 2018). (2) The oldest known *Cryptotis* dates back about 9 million years ago (mean = 0, stdve = 1, offset = 9 million years ago) (Harris, 1998). (3) The oldest *Otisorex* dates back about 3.5 million years ago (mean = 0, stdve = 1, offset = 3.5 million years ago) (Maldonado et al., 2001). The rest of the parameters were set as in the phylogenetic analyses.

We used Network v4.5 to construct phylogenetic trees of each nuclear gene and mitochondrial gene CYT B. Before building the tree, we phased our data in DnaSP v5.10 (Librado & Rozas, 2009; Stephens & Donnelly, 2003), then used the haplotype to construct the median‐joining network (Bandelt et al., 1999), and finally used the maximum parsimony strategy to select the optimal phylogenetic tree (Polzin & Daneshmand, 2003).

We explored the population genetic structure in STRUCTURE 2.3.1 (Pritchard et al., 2000) based on nDNA data. The number of genetic clusters (K value) was estimated by the admixture model with correlated allele frequencies. We set the K value from 1 to 10 to find the best value for K. Structure was run with 400,000 after 100,000 runs as burn‐in. Structure results were based on 10 independent runs. We got the results from Structure Harvester (https://taylor0.biology.ucla.edu/structureHarvester/).

### 2.3 Morphological analyses

We obtained 33 skulls from adult individuals. All specimens were deposited at Sichuan Normal University (SCNU), Sichuan Academy of Forestry (SAF), and Sichuan University (MSCU). Three external measurements— namely, head and body length (HB), tail length (TL), and hind foot length (HL) were measured in the field or recorded from original specimen labels. However, they were not used for morphological analyses because these measurements may show considerable inter‐observer variation (Jiang et al., 2003). Fourteen craniomandibular variables were measured using a digital caliper graduated to 0.01 mm. These were condyloincisive length (CIL), braincase height (BH), braincase breadth (BB), interorbital breadth (IOB), rostral length (RL), post‐rostral length (PRL), post palatal length (PPL), rostral breadth (RB), palatoincisive length (PIL), upper tooth row length (UTRL), maximum width across upper second molars (M^2^‐M^2^), lower tooth row length (LTR), mandibular length (ML), and length of the lower incisor (i1L). The measuring methods were following Jiang and Hoffman (2001), Woodman and Timm (1993), and Yang et al. (2007). Measurements of each specimen are shown in Table S4.

The numerical analyses were performed using SPSS v20.0 (SPSS Inc., Chicago, 154 IL, USA) (George & Mallery, 2011). A one‐way ANOVA was used to calculate the variables' means and standard deviations (SD) of all skull measurements. Variances of different variables between groups were also tested using an independent‐sample *t* test. We used 32 of the 33 relatively complete skulls to analyze morphometric variation using craniomandibular variables by a principal component analysis (PCA). The overall variables were log_10_‐transformed before conducting the PCA, and a few missing values were replaced by means. On the basis of the results of our molecular analyses, we assigned two specimens of *N. elegans* from Tibet (Markam) (see Section 3).

## 3 RESULTS

### 3.1 Phylogenetic relationships and genetic distance

**FIGURE 2:**
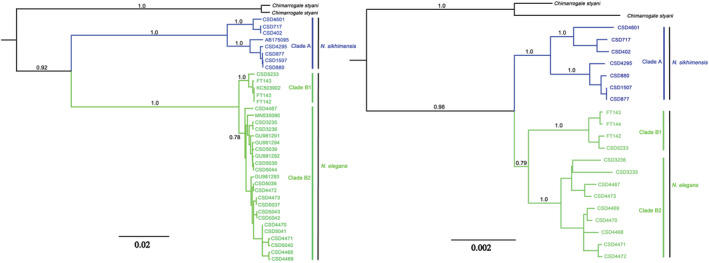
Results of Bayesian phylogenetic analyses of mtDNA dataset (left) and nDNA dataset (right). Numbers above branches refer to Bayesian posterior probabilities (PP). Branch lengths represent substitutions per site.

We obtained 29 CYT B sequences of 1128 bp and 22 nuclear gene sequences of 5055 bp. The new sequences were deposited in GenBank (Accession numbers ON160936–ON1601124, ON219777–ON219792, Table S1). For the two datasets, the results of phylogenetic analyses estimated by RAxML and BEAST were highly similar to each other, and only the Bayesian Inference (BI) gene trees are shown in Figure 2.

The trees obtained by BEAST analyses and ML analyses based on the mitochondrial gene supported the monophyly of the genus *Nectogale* (PP = 0.92, BS = 100) and the genus was divided into two clades (clade A and clade B). Clade A comprised eight specimens from southeast QTP (PP = 1.0, BS = 97). The clade B (PP = 1.0, BS = 97), including all sequences from Sichuan, Yunnan, Qinghai, and two sequences from Tibet (Markam), showed differentiated tree topologies in the BI tree and ML tree. Clade B was divided into two subclades (B1 and B2) in the BI tree but not in the ML tree (Figure S1). In our nuclear gene tree, a similar topology was recovered in the mtDNA tree (BI), but there were still differences (e.g., the monophyly of clade B was poorly supported [PP = 0.79, BS = 34]; mtDNA gene tree and nDNA gene tree have very different branch lengths).

The K2P genetic distances of CYT B among the 29 samples in the genus *Nectogale* ranged from 0% to 14.9%, with an average of 5.5%. The genetic distance within each clade was ≤1.63% (Table 2). The average genetic distance between clades B1 and B2 was the smallest, only 1.1%. The other average genetic distances were 14.33% (clades A and B1) and 14.52% (clades A and B2), which were close to the average genetic distance between *N. elegans* and *C. styani* (17.5%).

**TABLE 2:** Kimura 2‐parameters (K2P) genetic distances in the genus *Nectogale*, and between *Chimarrogale* and *Nectogale* species based on the CYT B gene

Average genetic distance (%)	Clade A	Clade B1	Clade B2	*C. styani*
Clade A	1.63			
Clade B1	14.33	0.21		
Clade B2	14.52	1.10	0.39	
*C. styani*	17.93	17.25	17.71	0.62

### 3.2 Divergence time

**FIGURE 3:**
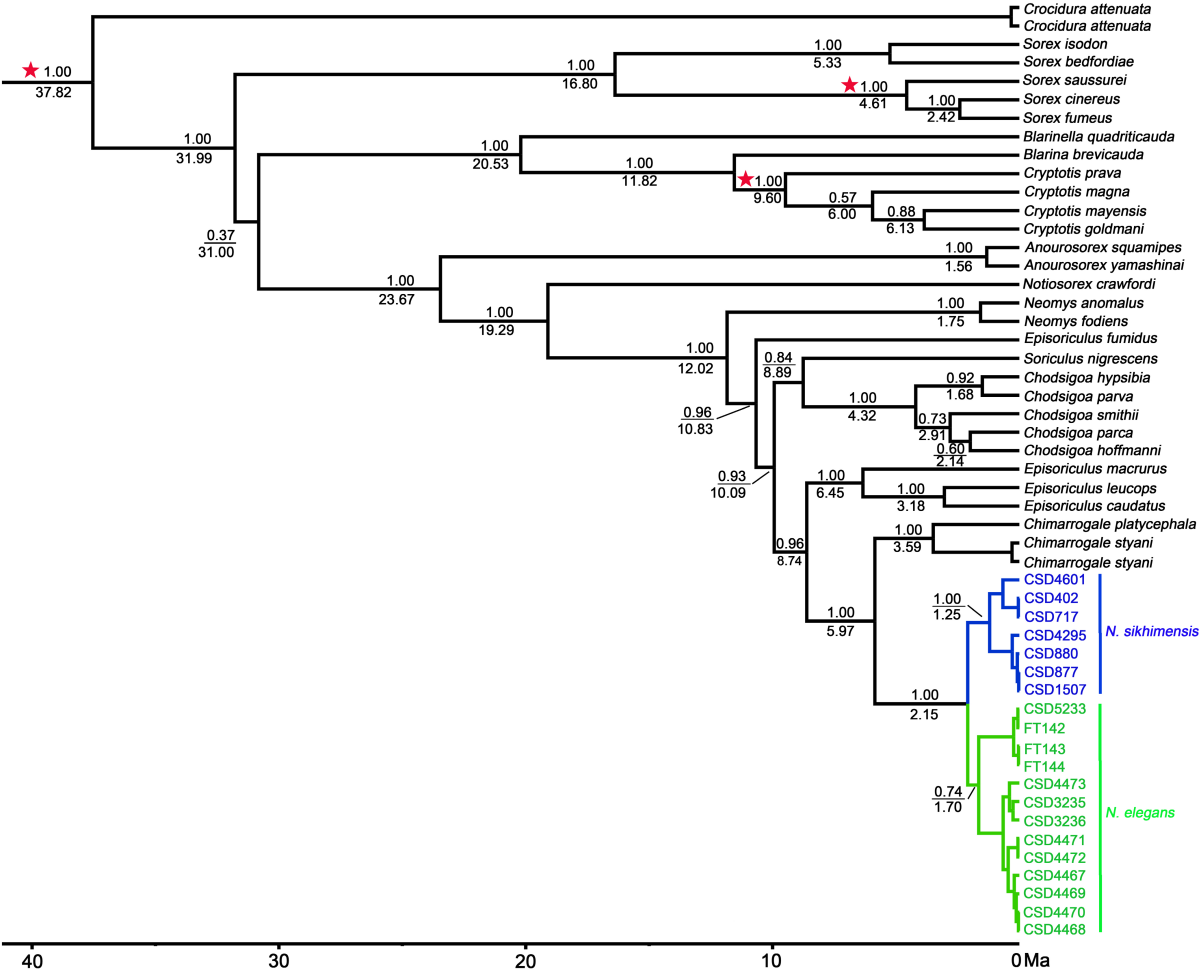
Divergence times estimated using BEAST based on nDNA dataset. Branch lengths represent time. Numbers above the nodes indicate posterior probabilities (PP). Numbers below the nodes represent the median divergence time. The three red asterisks indicate fossil‐calibrated nodes.

The Bayesian analysis of the divergence time tree showed the same phylogenetic relationship of *Nectogale* as the mitochondrial and nuclear gene tree (Figure 3). The divergence time between *Nectogale* and *Chimarrogale* was estimated to be approximately 5.97 million years ago (95% confidence interval [CI] = 3.79–8.35 million years ago). *N. elegans* and *N. sikhimensis* diverged from their common ancestors at approximately 2.15 million years ago (95% CI = 1.22–3.18 million years ago).

### 3.3 Mitochondrial and nuclear gene networks and population genetic structure

**FIGURE 4:**
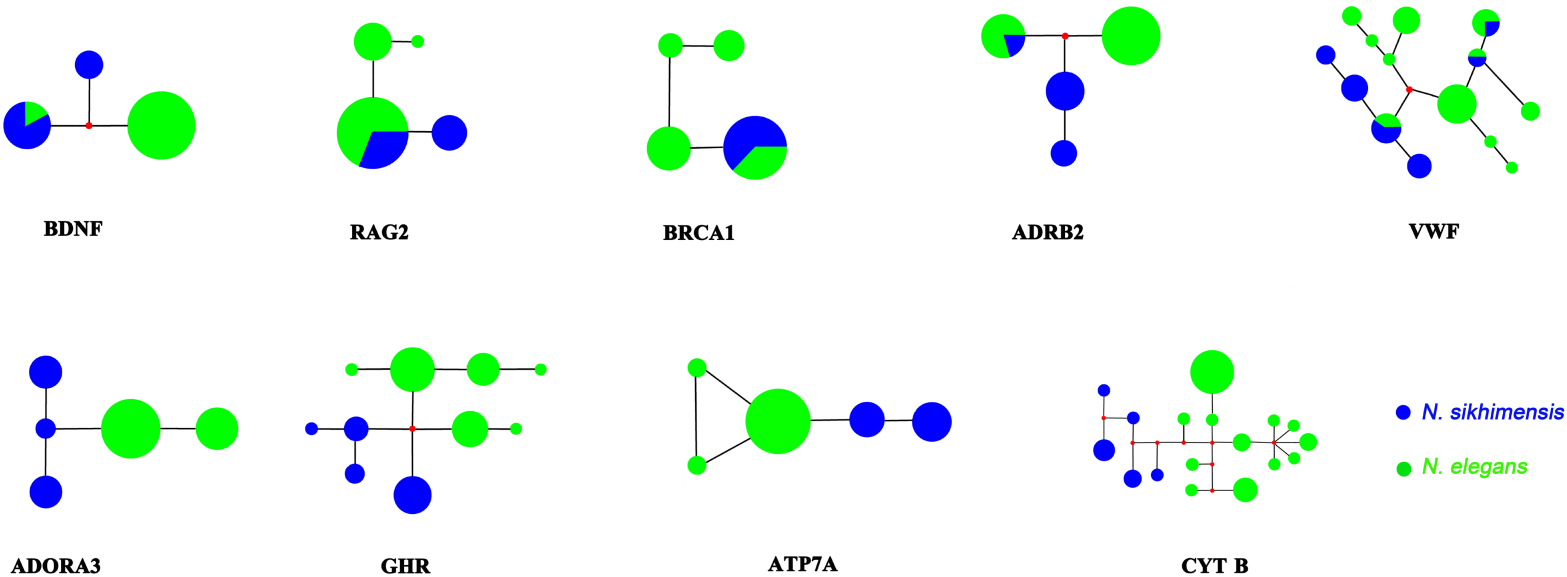
Median‐joining network based on mitochondrial and nuclear genes. Each circle represents a single haplotype scaled by its frequency; red dots represent missing or non‐sampled haplotypes.

Sequence characteristics of each gene used in network analyses are presented in Table 3. There were 17 haplotypes in the mitochondrial CYT B gene, and haplotype diversity (Hd) was 0.864. In nuclear genes, the haplotype diversity of VWF (0.919) and GHR (0.855) was higher than that in the other six nuclear genes (Table 3). The network analyses based on the nuclear gene (ADORA3, GHR, and ATP7A) showed that *N. sikhimensis* and *N. elegans* did not share haplotypes. However, haplotypes of the other nuclear genes (BDNF, RAG2, BRCA1, ADRB2, and VWF) were shared between *N. sikhimensis* and *N. elegans* (Figure 4).

**TABLE 3:** Sequence characteristics of samples used in the present study

Locus	CYT B	RAG2	ADORA3	ADRB2	BRCA1	GHR	ATP7A	VWF	BDNF
H	17	4	5	4	4	10	5	14	3
S	175	4	4	7	3	14	7	19	4
Hd	0.876	0.537	0.740	0.703	0.629	0.855	0.646	0.919	0.554
Pi	0.049	0.001	0.004	0.004	0.003	0.005	0.004	0.005	0.003

Abbreviations: H, Number of haplotypes; Hd, Haplotype diversity; pi, Nucleotide diversity; S, Number of variable sites.

The plot for ∆*K* and *K* revealed peaks at *K* = 2, 4, and 9 (Figure S2A). The *L*(*K*) mean values showed the highest probability of ln for *K* = 4, after which it stabilized (Figure S2B). Combining the results of *L*(*K*) and ∆*K*, the best number of clusters in STRUCTURE was 2 and 4 (Figure S2C). At *K* = 2, the structure analyses identified two clades in the genus *Nectogale* (Figure S2), a result that was consistent with the phylogenetic analysis based on the nuclear gene (Figure 2). At *K* = 4, the entire population was divided into four clusters corresponding to the four main sample localities (Zayü of Tibet, Medog of Tibet, Yunnan, and Sichuan).

### 3.4 Morphological analyses

The skull measurements are given in Table 4. There were significant differences when all individuals in the two clades were subjected to an independent‐sample *t* test for each variable (Table 5). Except for five pairwise comparisons (BB, PRL, PPL, M^2^–M^2^, and i1L between the two clades, *p* = .063, .143, .075, .113, and .173, respectively), the other nine comparisons were significantly different.

**TABLE 4:** Craniomandibular measurements (mm) of *Nectogale* species in the present study, including mean values, standard deviations (top line), range, and sample size (bottom line).

Measurements	*N. Sikhimensis*	*N. elegans*
CIL	24.87 ± 0.62	26.17 ± 0.88
	24.27–25.46; 4	24.97–28.03; 24
BH	8.34 ± 0.21	8.8 ± 0.29
	8.1–8.57; 4	8.16–9.41; 24
BB	14.97 ± 0.51	15.55 ± 0.56
	14.32–15.42; 4	14.43–16.5; 26
IOB	5.89 ± 0.16	6.28 ± 0.34
	5.71–6.05; 4	5.57–6.89; 26
RL	10.59 ± 0.38	11.35 ± 0.41
	10.1–11.01; 4	10.61–12.25; 27
PRL	13.47 ± 0.32	13.95 ± 0.62
	13.13–13.91; 4	13.12–15.2; 26
RB	7.89 ± 0.39	8.48 ± 0.32
	7.47–8.29; 4	7.87–9.07; 28
PPL	8.97 ± 0.34	9.43 ± 0.48
	8.47–9.26; 4	8.71–10.45; 24
PIL	11.98 ± 0.53	12.78 ± 0.5
	11.31–12.61; 4	11.85–13.91; 27
UTRL	11.14 ± 0.38	11.79 ± 0.41
	10.61–11.52; 4	10.98–12.63; 27
M^2^–M^2^	7.21 ± 0.42	7.48 ± 0.3
	6.7–7.67; 4	7.06–8.08; 28
ML	15.9 ± 0.48	16.74 ± 0.55
	15.24–16.37; 4	15.73–17.9; 28
LTR	10.19 ± 0.38	10.8 ± 0.37
	9.71–10.61; 4	10.07–11.57; 28
i1L	4.98 ± 0.45	5.38 ± 0.23
	4.54–5.47; 4	4.84–5.88; 28

**TABLE 5:** Independent‐sample *t* test of different variables within genus *Nectogale* and factor loadings, eigenvalues, and percentage of variance explained for principal component analysis.

Variables	*N. Sikhimensis*–*N. elegans*		PC1	PC2
	*t*	*p*		
CIL	2.820	.009*	0.957	−0.126
BH	3.091	.005*	0.680	−0.290
BB	1.940	.063	0.899	−0.308
IOB	2.243	.033*	0.895	−0.142
RL	3.498	.002*	0.934	0.217
PRL	1.507	.143	0.875	−0.323
RB	3.311	.002*	0.801	−0.300
PPL	1.858	.075	0.836	−0.376
PIL	2.999	.006*	0.961	0.185
UTRL	2.971	.006*	0.916	0.343
M^2^‐M^2^	1.634	.113	0.862	0.038
ML	2.914	.007*	0.964	0.106
LTR	3.092	.004*	0.884	0.401
i1L	1.747	.173	0.535	0.698
Eigenvalues			10.470	1.410
Variance explained (%)			74.785	10.074

* *p* < .05.

**FIGURE 5:**
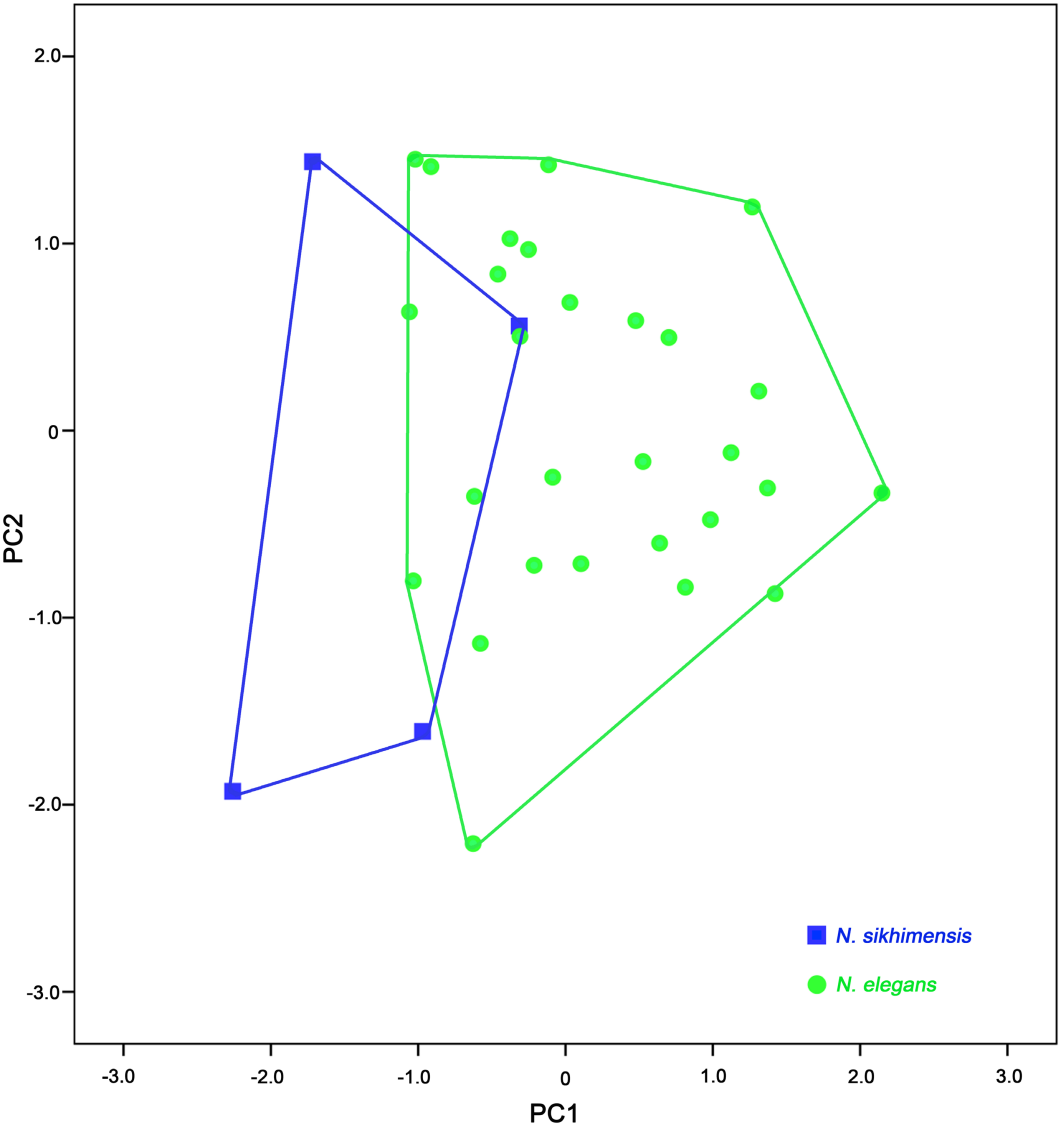
Results of principal component analysis of Nectogale based on the 14 craniodental measurements.

The results of the PCA analysis based on 14 craniodental measurements showed that the first two principal component eigenvalues were all greater than 1, accounting for 84.859% of the total variation. The first principal component (PC1) accounted for 74.785% of the total variance and was positively correlated with all variables (Table 5). Loadings related to skull length are ≥ 0.9, including condyloincisive length (CIL), rostral length (RL), palatoincisive length (PIL), upper tooth row length (UTRL), and mandibular length (ML). Loadings related to the width of the skull are ≥0.8, including the braincase breadth (BB), interorbital breadth (IOB), rostral breadth (RB), and maximum width across the upper second molars (M^2^–M^2^). The first principal component was highly correlated with these characters, and therefore, this component reflected a size effect. The second principal component (PC2) accounted for 10.074% of the total variation and was highly positively correlated with the length of the lower incisor (i1L) (loading >0.6). On the PC1 and PC2 plot (Figure 5), *N. sikhimensis* came from southern Tibet and occupied the negative region of PC1, *N. elegans* partially overlaps with *N. sikhimensis*.

**FIGURE 6:**
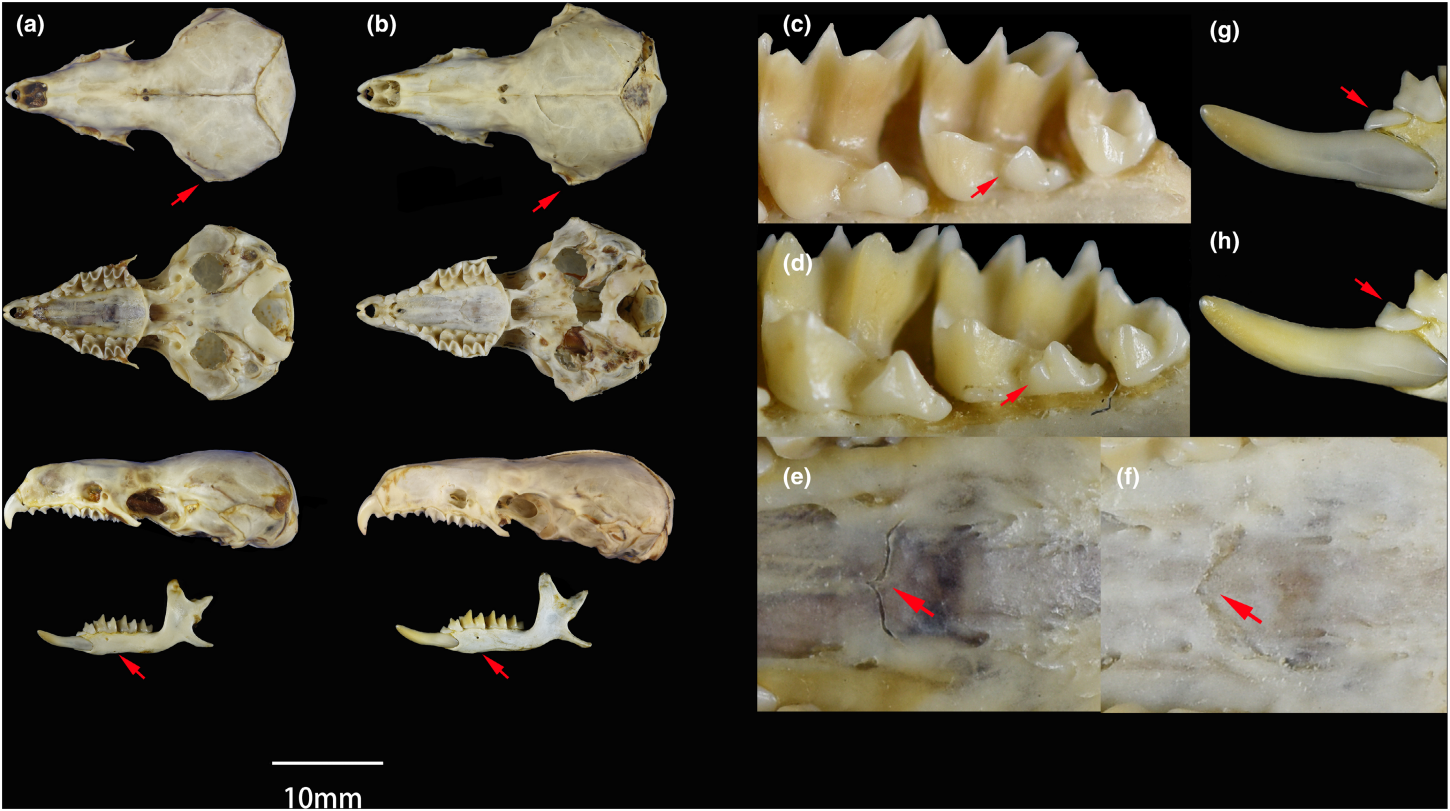
Skulls, mandibles, lingual view of the left second upper molar (M2), the palatal suture, and the cusp of lower unicuspid (a1) of *N. sikhimensis* (SAF07987; a, c, e, g) and *N. elegans* (SAF06144; b, d, f, h).

*N. sikhimensis* differs from *N. elegans* in its generally smaller skull, the average CIL (24.87 mm) is shorter than the later (26.17 mm). In addition, some characters that could distinguish these two clades were found. For example, the mastoid does not extend laterally in *N. sikhimensis* (Figure 6a), but in *N. elegans* the mastoid extends laterally, resulting in a more obviously dorsal view of the skull (Figure 6b). In *N. sikhimensis*, a cusp rests in the posterior of the hypocone and no cusp in the anterior of M^2^ in lingual view (Figure 6c), but in *N. elegans* there is a cusp in both the anterior and posterior of the hypocone of M^2^ (Figure 6d). In *N. sikhimensis*, the palatal suture is arcuate‐shaped (Figure 6e), but in *N. elegans*, the palatal suture is angle‐shaped formed by two intersecting straight lines (Figure 6f). Differences also exist in the mandible. In *N. sikhimensis*, the first half of the horizontal ramus of all specimens from Yunnan and most specimens from Sichuan is stouter than in *N. elegans* (Figure 6a, b). In *N. sikhimensis*, the tip of the a_1_ is shorter than in *N. elegans* resulting in a smaller appearance (Figure 6g, h).

## 4 DISCUSSIONS

Many studies have shown a high level of species diversity in the tribe Nectogalini. For example, two new species of the genus *Chodsigoa* (Kastschenko, 1907) were recently described (*C. hoffmanni* and *C. dabieshanensis*) (Chen et al., 2017, 2022). Some subspecies, such as *Chodsigoa furva* (Anthony, 1941), *Chimarrogale leander* (Thomas, 1902), and *Neomys milleri* (Mottaz, 1907) were recommended species status (Burgin & He, 2018; Chen et al., 2017; Igea et al., 2015; Yuan et al., 2013). New genera may exist in the tribe. For example, *Episoriculus fumidus* (Thomas, 1913) was suggested to be attributed to a new genus (Abramov, Bannikova, Chernetskaya, et al., 2017; He et al., 2010), and the genus *Chimarrogale* was proposed to be divided into two separate genera [*Chimarrogale* and *Crossogale* (Thomas, 1921)] by Abramov, Bannikova, Lebedev, and Rozhnov (2017).

Within the genus *Nectogale*, *N. sikhimensis* was first described from Sikkim based on differences in fur and teeth from *N. elegans*, but no molecular studies were performed to date to evaluate the genetic divergence of these two species. In this study, the results of phylogenetic trees based on mitochondrial and nuclear indicated that *Nectogale* was divided into two clades. Clade A is representing *N. sikhimensis*, clade B is *N. elegans*. Given the large genetic distance and morphological differences between the two taxa, we recover species status of *N. sikhimensis* and support that the genus *Nectogale* consists of two species. It is worth noting that the results of the structure analyses revealed that the species of the genus *Nectogale* were divided into two clusters (*K* = 2, corresponding to *N. sikhimensis* and *N. elegans*) or four clusters (*K* = 4, corresponding to four main sample localities of specimens). The results are very interesting, especially because this could indicate that cryptic diversity might be present. The results may be important to provide additional insights for future work.

The type locality of *N. elegans* is in Baoxing, Sichuan, which was also found in Yunnan, Shaanxi, Gansu, and Qinghai, China. On the basis of our collection, it is also distributed in Markam, eastern Tibet of China (Figures 1 and 2). The type locality of *N. sikhimensis* is Sikkim (India). This species is mainly distributed in southeast QTP of China and was also found in Bhutan, Myanmar, and Nepal (Smith & Xie, 2009). It seems that Mt. Gaoligong and Mt. Boshulaling are the boundaries between the two species.

The Eastern and southern parts of the Qinghai–Tibet Plateau region is one of the most important biodiversity hotspots in the world (Myers et al., 2000), where several new species have been discovered. For instance, two new species [*Neodon medogensis* (Liu et al., 2017) and *Alpiscaptulus medogensis* (Chen et al., 2021)], and a cryptic species of the white‐toothed shrews (*Crocidura* sp. 3) (Chen et al., 2020) were discovered in Medog of Tibet. In addition, *Neodon linzhiensis* (Liu et al., 2012)*, Neodon nyalamensis* (Liu et al., 2017)*, Niviventer fengi* (Ge et al., 2021), and *Eupetaurus tibetensis* (Jackson et al., 2021) were discovered in Gongbujiangda of Tibet, Nyalam of Tibet, Jilong of Tibet, and Gyantse of Tibet, respectively. In addition, molecular analyses identified high genetic diversity in birds (*Garrulax*) (Qu et al., 2011), rodents (*Apodemus*) (Fan et al., 2012), pikas (*Ochotona*) (Koju et al., 2017), and insectivores (*Sorex*, *Crocidura*, *Scaptonyx*) (Chen et al., 2015, 2020; He et al., 2019). The complex topography (the huge mountains and deep rivers) and geographic history (the uplift of QTP), along with diversified climate conditions may be relevant to the high species diversity and genetic diversity.

Diversification of the genus *Nectogale* may have been driven by the uplift of QTP. The divergence time between *N. elegans* and *N. sikhimensis* dates back to approximately 2.15 million years ago, corresponding to the most recent uplift of QTP, namely, 3.6–1.7 million years ago (Li et al., 1996). At that time, the QTP occurred a dramatic uplift (Li et al., 1996; Li & Fang, 1999), which changed the topography and habitats of the surrounding area and may thus be driving the diversification and evolution of *Nectogale*. The role of geographic isolation in speciation and diversification has been demonstrated in previous studies (Qu et al., 2014; Xing & Ree, 2017). Although rivers are not barriers for water shrews (Yuan et al., 2013), the huge bulging mountains (Mt. Boshulaling, Mt. Taniantaweng, and Mt. Mangkang) may act as a barrier to hinder gene flow between *Nectogale* species.

Notably, there is a discrepancy in divergence time between our study and He et al. (2010) who thought the Nectogalini radiated at 6.63 million years ago and the divergence between *Chimarrogale* and *Nectogale* dated back to 3.71 million years ago. In this study, the divergence dated back to 12.02 million years ago (Nectogalini) and 5.97 million years ago (*Chimarrogale* and *Nectogale*). The difference in calibration information may be the main reason for the discrepancy. According to recent studies, the division of Soricinae and Crocidurinae occurred at 36 million years ago (He et al., 2018; Springer et al., 2018), instead of 20 million years ago (He et al., 2010; Reumer, 1994).

This discordance between mitochondrial and nuclear gene trees has been observed in our study. For example, the two subclades B1 and B2 were strongly supported in mtDNA gene trees but not in nDNA trees. This discordance often was attributed to several factors, i.e., mitochondrial capture (Dong et al., 2014), explosive speciation (Krause et al., 2008), incomplete lineage sorting (Edwards, 2009), and introgression (Funk & Omland, 2003; Yannic et al., 2010). The most likely reason for cyto‐nuclear incongruence is that the mitochondrial gene CYT B was captured during the divergence of the subclades B1 and B2. This process has been observed in many cases (e.g., Bryson et al., 2010; Dong et al., 2014; Markova et al., 2013; Tang et al., 2012).

## 5 CONCLUSIONS

In the present study, we used mitochondrial and nuclear genes to investigate the phylogenetic relationships and evolutionary history of the genus *Nectogale*. The results of molecular analyses supported the division of the genus into two clades (Clade A = *N. sikhimensis*, Clade B = *N. elegans*). Furthermore, several diagnostic characteristics were found in these two clades. Therefore, the molecular and morphological evidence supported that the genus *Nectogale* consists of two valid species: *N. sikhimensis* and *N. elegans*. In addition, our divergence time tree suggested that the split of *Nectogale* species might be relevant to the QTP uplift.

## AUTHOR CONTRIBUTIONS

**Ronghui Fan:** Formal analysis (lead); investigation (lead); methodology (lead); software (lead); writing – original draft (lead); writing – review and editing (equal). **Keyi Tang:** Data curation (equal); formal analysis (equal); methodology (equal); writing – review and editing (lead). **Liang Dou:** Data curation (equal); investigation (equal); resources (equal). **Changkun Fu:** Formal analysis (equal); investigation (equal); methodology (equal). **Abu ul Hassan Faiz:** Investigation (equal); resources (equal). **Xuming Wang:** Formal analysis (equal); investigation (equal). **Yufan Wang:** Data curation (equal); investigation (equal); resources (equal). **Shunde Chen:** Formal analysis (equal); funding acquisition (lead); investigation (equal); methodology (lead); resources (lead); supervision (equal); writing – review and editing (lead). **Shaoying Liu:** Data curation (equal); formal analysis (equal); investigation (equal); methodology (equal); resources (lead); supervision (lead); writing – review and editing (lead).

## CONFLICT OF INTEREST

The authors declare that they have no conflict of interest.

## Supporting information


Figure S1

Figure S2

Table S1

Table S2

Table S3

Table S4

## Data Availability

New DNA sequences in this study were deposited in GenBank (Accession numbers ON160936–ON161124, ON219777–ON219792). (https://www.ncbi.nlm.nih.gov/).
