# Supplementary figures and images for: Molecular phylogeny and taxonomy of the genus Nectogale (Mammalia: Eulipotyphla: Soricidae)
Source: Ecol Evol. 2022 Oct 13;12(10):e9404. doi: 10.1002/ece3.9404 (PMC9596327; doi:10.1002/ece3.9404)

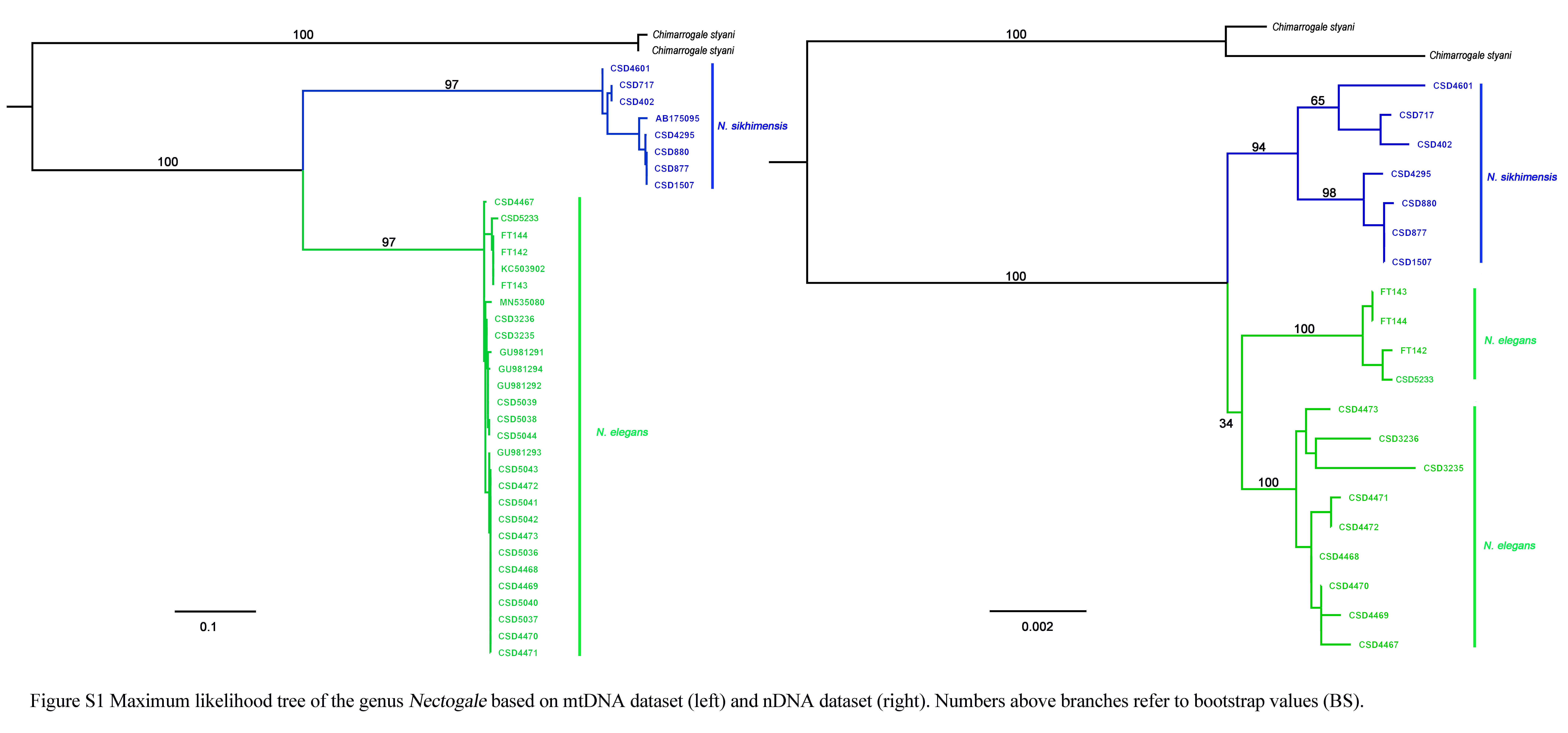

Supplement: Supplementary file 1 — Figure S1 [file ECE3-12-e9404-s003.jpg]

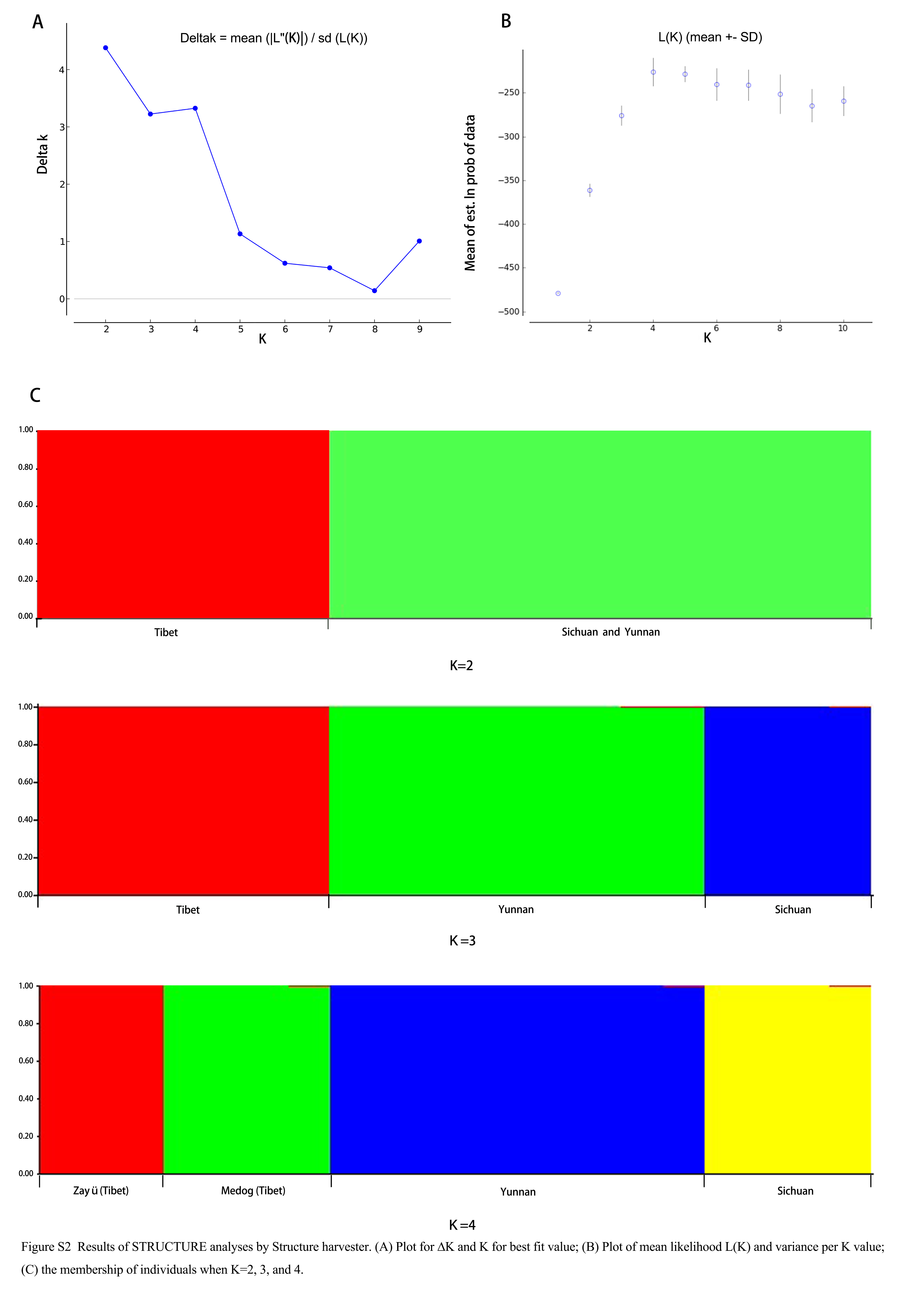

Supplement: Supplementary file 2 — Figure S2 [file ECE3-12-e9404-s002.jpg]
